# Common and novel haplotype structures between different types of cancer

**DOI:** 10.1002/cnr2.2107

**Published:** 2024-06-21

**Authors:** Morteza Gholami

**Affiliations:** ^1^ Department of Paramedicine, Amol School of Paramedical Sciences Mazandaran University of Medical Sciences Sari Iran; ^2^ Metabolic Disorders Research Center, Endocrinology and Metabolism Molecular‐Cellular Sciences Institute Tehran University of Medical Sciences Tehran Iran

**Keywords:** expression, genome‐wide association study, haplotype, lncRNA, variant

## Abstract

**Background:**

Background: Genome‐wide association studies (GWAS) have identified hundreds of genetic variants associated with cancer risk. GWAS data are important for cancer prevention and understanding the underlying mechanisms of cancer.

**Aims:**

This study aimed to investigate the genetic association between different types of cancer using GWAS data and a bioinformatics approach.

**Methods and results:**

The significant GWAS variants associated with more than one cancer type were identified. Common linkage disequilibrium (LD) variants between different types of cancer were identified by 1000 genomes phase 3 LD data. Haplotype blocks were identified by analyzing 1000 Genomes phase 3 genotyping data in the GWAS populations. Subsequent analyses included functional SNP analyses and TCGA gene expression. The results associated with significant GWAS variants (P<5E‐8) showed the following haplotype associations in European population: GT rs4808075‐rs8170 haplotype on BABAM1 with breast and ovarian cancers, GC rs16857609‐rs11693806 haplotype on DIRC3 with breast and thyroid cancers, GCG rs380286‐rs401681‐rs31487 haplotype on CLPTM1L with skin and lung cancers, GGG rs4430796‐rs11651052‐rs11263763 haplotype on HNF1B with prostate and endometrial cancers, and GT rs10505477‐rs6983267 haplotype on CASC8 associated with colorectal and prostate cancers. All these genes had significantly different expressions in tumor tissues (P<1E‐3). In addition, the rs11693806 variant is located in the hsa‐miR‐873‐5p binding site and has an enhancing effect on the hsa‐miR‐873‐5p:DIRC3 interaction.

**Conclusion:**

These novel haplotype structures and miRNA:lncRNA interactions are important for understanding the common genetic link between cancers. These results can potentially be used in genetic panels.

## 1 INTRODUCTION

Based on cancer statistics, 2022 cancer deaths and overall incidence have been averted since 1991, but it is still the second leading world cause of death in the world after heart disease.^1^ The decrease in cancer mortality is associated with reduced smoking, cancer risk factors, and progress in screening tests, diagnosis, and treatment.^1^, ^2^ However, the incidence of cancer worldwide is expected to increase in the coming decades because of the influence of demographic factors such as population growth and aging, and is predicted to double by 2020–2070.^3^ There are more than 200 types of cancer and many can be prevented or treated effectively if diagnosed early.^4^ After the outbreak of corona disease, the diagnosis and treatment of cancer were affected.^1^

As a multifactorial disease, different risk factors such as environmental factors, age, obesity, or genetics are effective for cancer. Some cancer incidence is related to inherited genetic factors. On the other hand, genetic changes related to cancer occur by mistake and based on various factors such as environmental factors. While knowledge of the genetics of cancers has improved and many genetic and epigenetic factors associated with cancer have been identified, there are still many unknown genetic factors.^5^, ^6^, ^7^ In recent years, genome‐wide association studies (GWAS) have investigated the role of genetic variants (genotype) on the risk of cancer (phenotype) and have found many variants and genes associated with cancer or interactions with drug treatment.^8^, ^9^ GWAS data can be used to identify clinical risk factors, underlying mechanisms, develop drug targets, and predict complex traits such as cancer.^10^, ^11^ However, the biological functions of many GWAS variants and their mechanisms of action remain unclear.

In this study, for the first time, we aimed to investigate all cancer‐associated GWAS variants to find common genetic bases and associations including variants, genes, and haplotypes between different types of cancer using 1000 genome phase 3, TCGA data, and bioinformatics approaches.

## 2 METHODS

### 2.1 Study pipeline

**FIGURE 1:**
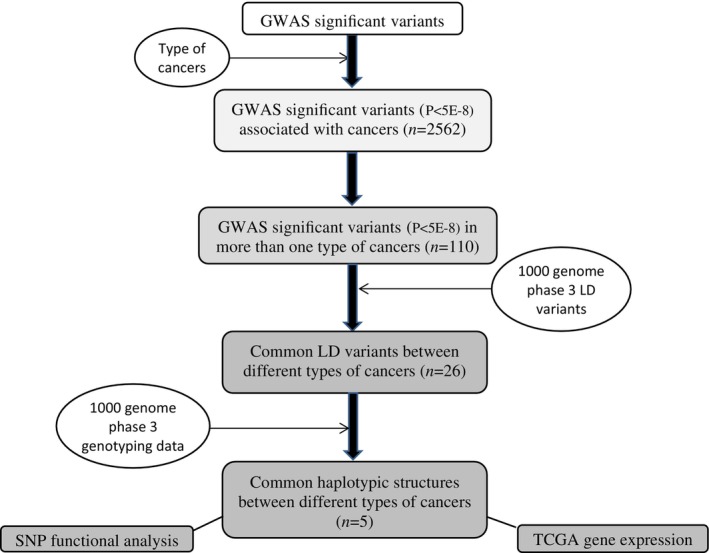
Study pipeline.

GWAS significant variants associated with cancers were obtained from all GWAS significant variants. Then GWAS significant variants were determined in more than one type of cancer. The 1000 genome phase 3 LD variants were used to identify common linkage disequilibrium (LD) variants between different types of cancers and candidate haplotypic variants associated with GWAS‐significant LD variants. After that, SNP functional analysis of SNP and TCGA gene expression were performed on the results. The complete flowchart is shown in Figure 1.

### 2.2 Common GWAS LD variants

The GWAS catalog (gwas_catalog_v1.0.2_associations_e0_r) was downloaded from GWAS catalog‐EMBL‐EBI (https://www.ebi.ac.uk/gwas/) to find common GWAS variants. Significant GWAS variants associated with cancer types were identified using the names of cancers in the disease/trait section. These names are presented in Data S1. After that, significant variants were identified by combining cancer types based on variant names in the R programming language (version 4.2.1). For this purpose, we created a dataset for GWAS significant variants and GWAS traits in SAV format, and then we used the haven package and wrote a specific script to split data and combine them based on GWAS traits (Data S2). Finally, variants with more than one cancer type were identified by sorting the results.

The Ensembl genome browser 110 (https://asia.ensembl.org/index.html) LD calculator was used for GWAS significant variants to find LD variants with (D′ and *r* ^2^ ≥ .6) for all 1000 genome phase 3 populations. Then, to find haplotypic blocks between these significant GWAS LD variants in specific GWAS super‐populations, the 1000‐genome phase 3 data containing GWAS LD variants were downloaded from Ensembl Genome Browser 110 (https://asia.ensembl.org/index.html).^12^ Haplotypic blocks and LD plots were generated using HaploView V4.2.

### 2.3 SNP function

SNP function for gene expression was identified using the Genotype‐Tissue Expression (GTEx) portal (https://gtexportal.org/home/). Gene name, SNP position, and type of variant were investigated in dbSNP (https://www.ncbi.nlm.nih.gov/snp/).^13^ The variants in 3UTR and their flanking regions (+/− 25 nucleotides) were investigated for conserved miRNA binding sites using TargetScan (release 8: https://www.targetscan.org/vert_80/), dbSNP (https://www.ncbi.nlm.nih.gov/snp/) for variant location, and Galaxy (https://usegalaxy.org/) for finding whether the variant is located in a miRNA binding site.^13^, ^14^ For lncRNA genes, all GWAS significant variants and their LD variants in miRNA binding sites were identified by lncRNASNP v3 (http://gong_lab.hzau.edu.cn/lncRNASNP3/).^15^ We used MotifMap (https://motifmap.ics.uci.edu/#MotifSearch) to explore potential cancer‐specific motifs associated with the identified genes.^16^

### 2.4 Gene expression

The genes with haplotypic blocks associated with GWAS significant variants were included in gene expression analysis. In gene expression, up or downregulation of all TCGA tumor tissues were compared with normal tissues based on OncoDB (https://oncodb.org/index.html) and TIMER2.0 (http://timer.cistrome.org/) Gene_DE module via the Wilcoxon test. The results with *p*‐value <.001 were considered significant.^17^, ^18^

## 3 RESULTS

### 3.1 Common GWAS significant LD variants and genes between different types of cancer

The significant GWAS variants associated with cancers were identified from the GWAS catalog. There were 110 GWAS significant variants in more than one type of cancer (Data S3). Twenty‐six GWAS significant variants on eight genes were in LD and common between different types of cancers based on the 1000 genome phase 3. Most of them were intronic eQTL variants. The results are shown in Table 1. The DIRC3 is an lncRNA, and the rs11693806 variant was located in the hsa‐miR‐873‐5p:DIRC3 binding site (Table 2). None of the other GWAS significant and LD variants were located in the miRNA binding site (Data S4) or near any motifs associated with the identified genes (Data S5).

**TABLE 1:** GWAS significant LD variants (P<5E‐8) in more than one type of cancer.

Gene/variant/Chr:Position	Type of variant	Type of cancer	eQTL	LD variant variant/Chr:Position	Type of variant	Type of cancer	eQTL
CASC8							
rs6983267/8:127401060	Intron	Prostate cancer, Colorectal cancer		rs10505477/8:127395198	Intron	Prostate cancer, Colorectal cancer, Hormone‐sensitive cancer	
BABAM1(C19orf62)							
rs8170/19:17278895	Synonymous	Breast cancer, Ovarian cancer	Yes	rs4808075/19:17279482	Intron	Ovarian cancer, Cancer (pleiotropy)	Yes
DIRC3							
rs16857609/2:217431785	Intron	Breast cancer, Thyroid cancer	Yes	rs11693806/2:217427435	Exon	Breast cancer, Thyroid cancer	Yes
SLC22A3							
rs7758229/6:160419220	Intron	Colorectal cancer, Prostate cancer	Yes	rs12194182/6:160413483	Intron	Prostate cancer, Cancer (pleiotropy)	Yes
CLPTM1L							
rs380286/5:1320132	Intron	Skin cancer, Lung cancer, Adenocarcinoma in never smokers	Yes	rs401681/5:1321972	Intron	Bladder cancer, Lung cancer, Nasopharyngeal carcinoma, Lung adenocarcinoma, Pancreatic cancer, Skin cancer	Yes
				rs31487/5:1340986	Intron	Skin cancer, Lung cancer	
rs401681/5:1321972	Intron	Bladder cancer, Lung cancer, Nasopharyngeal carcinoma, Lung adenocarcinoma, Pancreatic cancer, Skin cancer		rs31490/5:1344343	Splice	Pancreatic cancer, Melanoma	
				rs31487/5:1340986	Intron	Skin cancer, Lung cancer	
rs380286/5:1320132	Intron	Skin cancer, Lung cancer, Adenocarcinoma in never smokers	Yes	rs31490/5:1344343	Splice	Pancreatic cancer, Melanoma	
rs31487/ 5:1340986	Intron	Skin cancer, Lung cancer		rs31490/5:1344343	Splice	Pancreatic cancer, Melanoma	
BRCA2							
rs11571818/13:32394673	Splice	Squamous cell lung carcinoma, Cancer(pleiotropy), Lung cancer		rs11571833/13:32398489	Stop	Lung cancer, Breast cancer	
HNF1B							
rs7501939/17:37741165	Intron	Testicular germ cell tumor, Prostate cancer	Yes	rs11263763/17:37743574	Intron	Prostate cancer, Endometrial cancer, Cancer (pleiotropy)	
				rs11651052/17:37742390	Intron	Endometrial cancer, Prostate cancer	Yes
rs4430796/17:37738049	Intron	Prostate cancer, Endometrial cancer, Hormone‐sensitive cancer		rs12601991/17:37741642	Intron	Cancer	
				rs7501939/17:37741165	Intron	Testicular germ cell tumor, Prostate cancer	
				rs11651052/17:37742390	Intron	Endometrial cancer, Prostate cancer	Yes
				rs11263763/17:37743574	Intron	Prostate cancer, Endometrial cancer, Cancer (pleiotropy)	
rs11651052/17:37742390	Intron	Endometrial cancer, Prostate cancer	Yes	rs11263763/17:37743574	Intron	Prostate cancer, Endometrial cancer, Cancer (pleiotropy)	
rs12601991/17:37741642	Intron	Cancer, Cancer (pleiotropy)		rs11651052/17:37742390	Intron	Endometrial cancer, Prostate cancer	Yes
TERT							
rs10069690/5:1279675	Intron	Ovarian cancer, Non‐glioblastoma glioma, Glioblastoma, Breast cancer, Chronic lymphocytic leukemia, Prostate cancer, Uterine leiomyoma		rs2242652/5:1279913	Intron	Prostate cancer, Breast cancer, Uterine leiomyoma, Hepatocellular carcinoma in alcohol related cirrhosis	
rs7725218/5:1282299	Intron	Cancer (pleiotropy), Prostate cancer		rs2853677/5:1287079	3'UTR	Lung cancer, Cancer, Skin cancer	Yes
				rs2736100/5:1286401	3'UTR	Lung cancer, Testicular germ cell cancer	Yes
				rs7705526/5:1285859	Intron	Ovarian cancer, Lung cancer, Cutaneous malignant melanoma, Chronic lymphocytic leukemia, Basal cell carcinoma	
rs7705526/5:1285859	Intron	Ovarian cancer, Lung cancer, Cutaneous malignant melanoma, Chronic lymphocytic leukemia, Basal cell carcinoma	Yes	rs2736100/5:1286401	3'UTR	Lung cancer, Testicular germ cell cancer	Yes
				rs2853677/5:1287079	3'UTR	Lung cancer, Cancer, Skin cancer	Yes
rs2736098/5:1293971	Synonymous	Pancreatic cancer, Prostate cancer, Breast cancer		‐	‐	‐	
rs2736100/5:1286401	3'UTR	Lung cancer, Testicular germ cell cancer		rs2853677/5:1287079	3'UTR	Lung cancer, Cancer, Skin cancer	Yes

**TABLE 2:** Effect of rs11693806 on hsa‐miR‐873‐5p:DIRC3 interaction.

miRNA	hsa‐miR‐873‐5p	chr9:28888925‐28888945(‐)
Gene	DIRC3	chr2:217284022‐217284863(‐)
TargetScan	Start: 217427432	End: 217427438
miRmap	Start: 217427432	End: 217427437
	ΔG duplex (kCal/mol)	−12.8
	ΔG binding (kCal/mol)	−12.94798
	ΔG open (kCal/mol)	15.63576
	TargetScan score	23.29224
	AU content	0.29224
	Exact probability	0.02779
	lncRNA:5' CAAGGAUAGGAUUUCCCAGACAUUCCUGAGGGAGAAAAU 3'	
	||||||.	
	miRNA:3' UCCUCUGAGUGUUCAAGGACG 5'	

### 3.2 Haplotypic blocks

**FIGURE 2:**
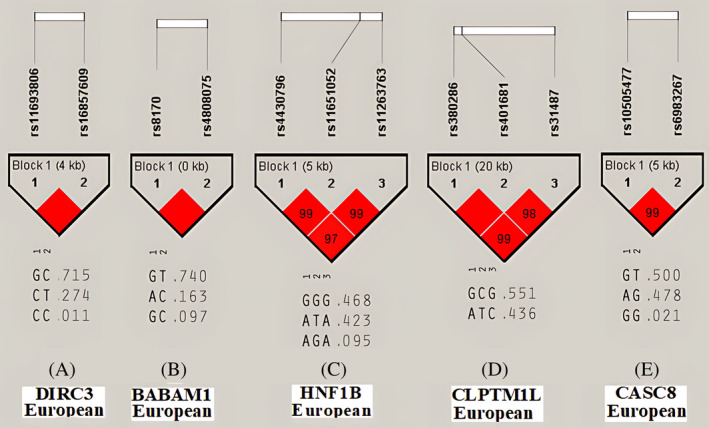
Haplotype blocks associated with GWAS significant variants. (A) DIRC3 haplotype; (B) BABAM1(C19orf62) haplotype; (C) HNF1B haplotype; (D) CLPTM1L haplotype; and (E) CASC8 haplotype. Results were related to European population (*n* = 503 subjects).

Further analysis of 1000 genome phase 3 genotyping data reveals candidate haplotypic blocks in five genes. The results are presented in Figure 2. HNF1B GGG haplotype in European population associated with prostate and endometrial cancers, CLPTM1L GCG haplotype in European population associated with skin and lung cancers, CASC8 GT haplotype in European populations associated with colorectal and prostate cancers, DIRC3 GC haplotype in European population associated with breast and thyroid cancers, and BABAM1(C19orf62) GT haplotype in European population associated with breast and ovarian cancers. Other results are shown in Data S6.

### 3.3 Gene expression based on TCGA data

**FIGURE 3:**
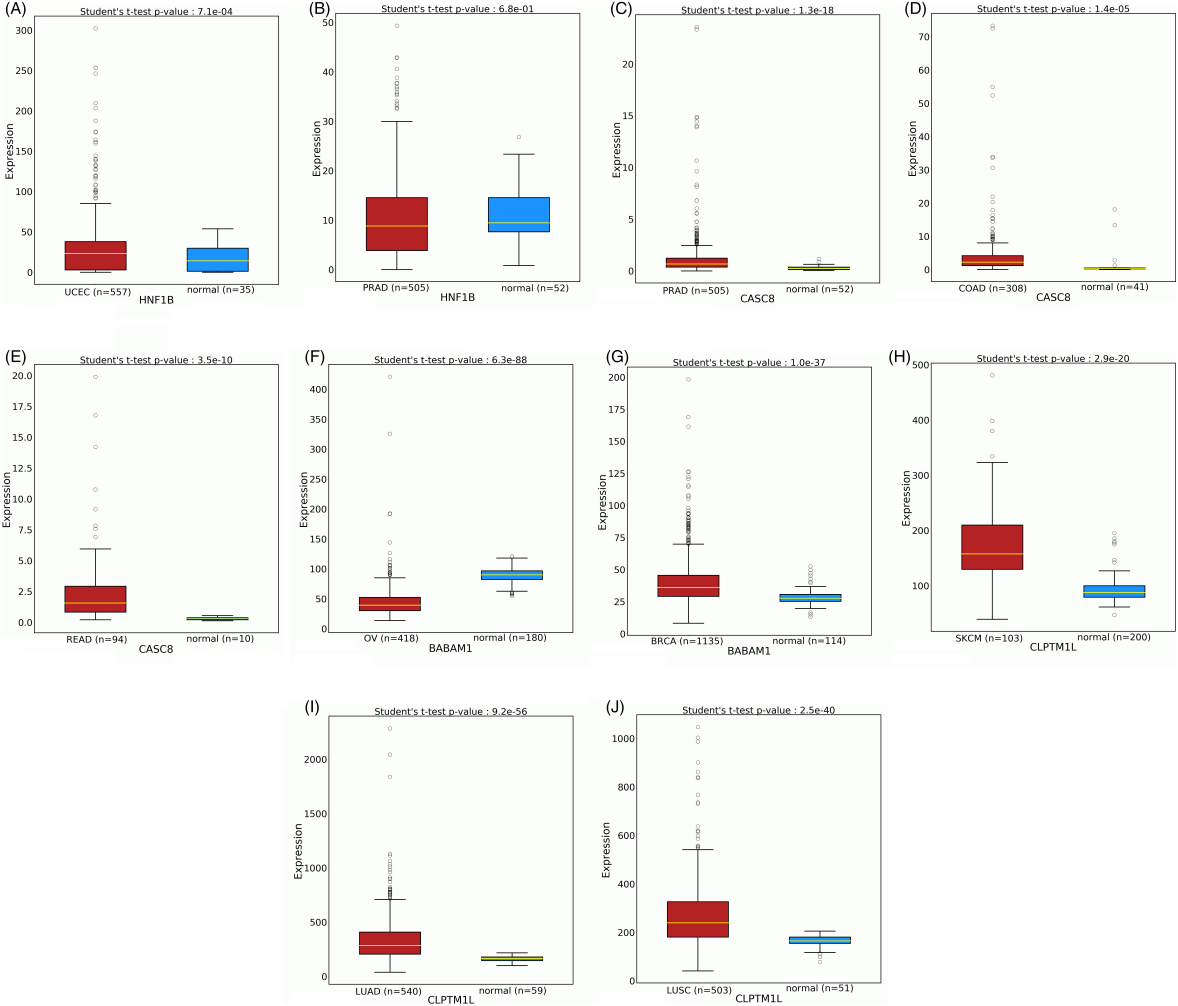
The expression of identified genes with haplotypic structures compared with normal tissues. (A) HNF1B for endometrial cancer; (B) HNF1B for prostate cancer (C) CASC8 for prostate cancer (D) CASC8 for colon cancer; (E) CASC8 for rectal cancer (F) BABAM1 for ovarian cancer; (G) BABAM1 for breast cancer (H) CLPTM1L for skin cancer; (I) CLPTM1L for lung adenocarcinoma; and (J) CLPTM1L for lung squamous cell carcinoma.

Based on the TCGA data, the expression of five genes with haplotypic structures was significantly different from normal tissues (P<1E‐3). The results for HNF1B, CASC8, BABAM1, and CLPTM1L, are shown in Figure 3. The DIRC3 results are shown in Data S7.

## 4 DISCUSSION

This study investigated the common role of GWAS significant genetic factors in different types of cancer. In this study, for the first time, the association of the 26 LD variants on eight genes with different types of cancer was identified. The rs11693806 variant of DIRC3 lncRNAs with a gain effect on hsa‐miR‐873‐5p:DIRC3 interaction located on hsa‐miR‐873‐5p biding site. The association of this variant with breast and thyroid cancer was identified in previous GWA studies, it may be related to the variant effect on hsa‐miR‐873‐5p and its target gene.^19^, ^20^, ^21^

Previous studies have investigated some genome‐wide haplotype associations, and new methods have explored the chromosome‐scale haplotype‐resolved reconstruction approach to characterize the cancer precise structural variant landscape.^22^, ^23^ However, in this study, for the first time, some haplotypic GWAS significant LD variants were identified for at least two types of cancer. The haplotypic structures were identified in DIRC3, BABAM1(C19orf62), HNF1B, CLPTM1L, and CASC8 genes. GT haplotypic structure of rs4808075‐rs8170 variants on BABAM1 gene is associated with both breast and ovarian cancers in European population. These two variants are located at a distance of 578 nucleotides from each other. The gene expression results showed that the BABAM1 gene expression is significantly different from adjacent normal tissues in breast and ovarian cancer. Thus, previous GWA studies separately identified the role of rs4808075 and rs8170 variants in breast and ovarian cancers.^24^, ^25^, ^26^, ^27^ Also, rs4808075 is a pleiotropic cancer susceptibility variant associated with five types of cancer, such as breast and ovarian cancer.^24^ GC haplotypic structure of rs16857609‐rs11693806 variants on DIRC3 gene is associated with breast and thyroid cancers in European population. These two variants are located at a distance of 4350 nucleotides from each other. The results of gene expression showed that DIRC3 gene expression is significantly different from adjacent normal tissues in breast and thyroid cancer. The role of rs16857609 and rs11693806 variants on breast or thyroid cancer has been investigated separately in previous studies.^19^, ^20^, ^28^, ^29^ In addition, the association between other DIRC3 variants with breast and thyroid cancer were investigated in a previous study.^30^ GCG haplotypic structure of rs380286‐rs401681‐rs31487 variants on CLPTM1L gene is associated with skin and lung cancers in European population. The gene expression results showed that CLPTM1L gene expression is significantly different from adjacent normal tissues in skin and lung cancers. The role of these variants in skin and lung cancers was investigated separately in previous GWA studies.^31^, ^32^, ^33^, ^34^ Other studies identified the association of rs401681variant with lung or skin cancers in other populations.^35^, ^36^ GGG haplotypic structure of rs4430796‐rs11651052‐rs11263763 variants with 5 kb distance on HNF1B gene is associated with prostate and endometrial cancers in European population. The gene expression results showed that HNF1B gene expression is significantly different from adjacent normal tissues in endometrial cancer. The associations of these variants with prostate and endometrial cancers had been confirmed by previous GWA studies.^37^, ^38^, ^39^, ^40^, ^41^, ^42^, ^43^, ^44^ Furthermore, the GT haplotypic structure of rs10505477‐rs6983267 variants with 5 kb distance on the CASC8 gene is associated with colorectal and prostate cancers in European population. The results of gene expression showed that CASC8 gene expression is significantly different from adjacent normal tissues in colorectal and prostate cancers. The role of rs10505477 and rs6983267 variants on colorectal and prostate cancers was investigated separately in previous studies.^29^, ^45^, ^46^, ^47^, ^48^, ^49^, ^50^, ^51^, ^52^, ^53^ Further studies identified the association of rs10505477 with colorectal and prostate cancers in other populations.^54^, ^55^

In conclusion, this study identified five novel haplotype structures and one miRNA:lncRNA interaction common between more than one type of cancer which is important for understanding the genetic mechanisms and association between cancers. These results can potentially be used in genetic panels.

## AUTHOR CONTRIBUTIONS

**Morteza Gholami:** Conceptualization; investigation; writing – original draft; writing – review and editing; methodology; software; formal analysis; supervision; data curation; validation.

## CONFLICT OF INTEREST STATEMENT

The author declares no conflict of interest.

## ETHICS STATEMENT

Not applicable.

## Supporting information


**Data S1.** Supplementary method 1.

**Data S2.** Supplementary method 2.

**Data S3.** Supplementary results 1.

**Data S4.** Supplementary results 2.

**Data S5.** Supplementary results 3.

**Data S6.** Supplementary results 4.

**Data S7.** Supplementary results 5.

## Data Availability

The data that supports the findings of this study are available in the supplementary material of this article.
