# Supplementary material for: Common and novel haplotype structures between different types of cancer
Source: Cancer Rep (Hoboken). 2024 Jun 21;7(6):e2107. doi: 10.1002/cnr2.2107 (PMC11190585; doi:10.1002/cnr2.2107)
Supplement: Supplementary file 2 — Data S2. Supplementary method 2. [file CNR2-7-e2107-s005.pdf]

```
library(haven)

data.all<-read_sav(choose.files())

Splited.data.all<- split(data.all , list(data.all$snp))

My.fun<- function(x){

if(dim(x)[1] != 1){

   x[,"traits.2"]<-paste0(do.call("c",  x[,"traits"]),collapse = ", ")

} else {

   x[,"traits.2"]<-as.character( x[,"traits"])

}

x}

My.fun.addnew<-lapply(Splited.data.all, My.fun)

xx<- My.fun.addnew[[2]][1,]

for (i  in 2:length(My.fun.addnew)) {

  xx<- rbind( xx, My.fun.addnew[[i]][1,])

}

xx<-xx[,-(1)]

write.table(xx,file="D:\\cancers gwas\\End.All9.txt",sep = "\t")
```
