# Supplementary figures and images for: Common and novel haplotype structures between different types of cancer
Source: Cancer Rep (Hoboken). 2024 Jun 21;7(6):e2107. doi: 10.1002/cnr2.2107 (PMC11190585; doi:10.1002/cnr2.2107)

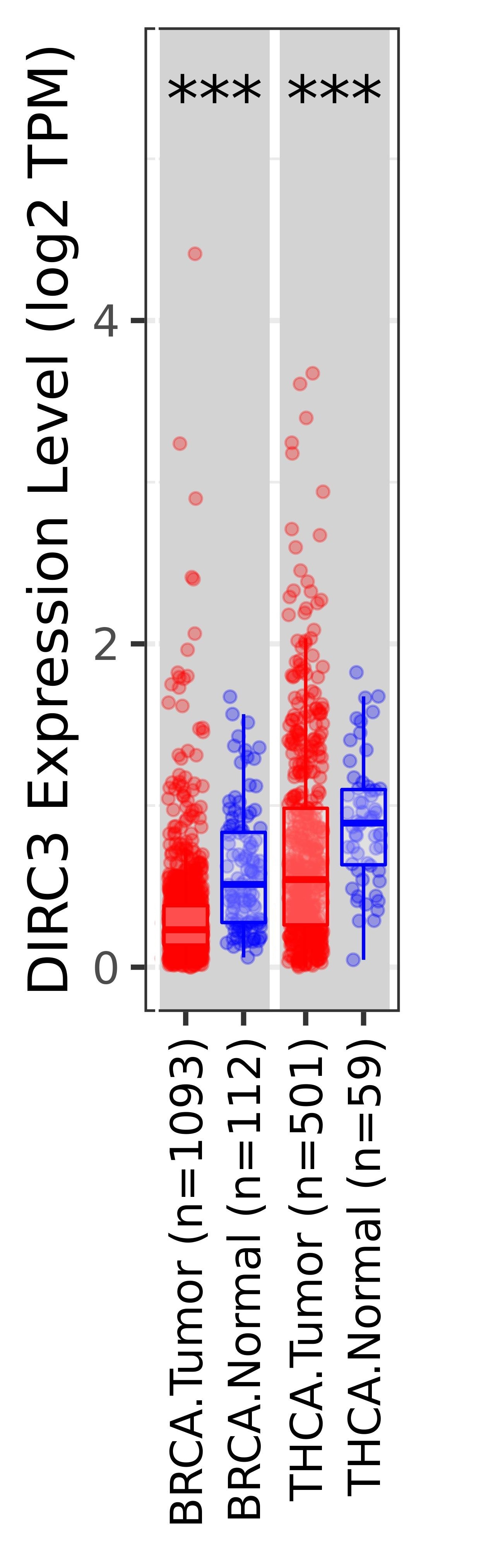

Supplement: Supplementary file 7 — Data S7. Supplementary results 5. [file CNR2-7-e2107-s006.jpg]
